# A Systematic Review and Meta-Analysis of Studies Comparing Concurrent Chemoradiotherapy With Radiotherapy Alone in the Treatment of Stage II Nasopharyngeal Carcinoma

**DOI:** 10.3389/fonc.2022.843675

**Published:** 2022-07-12

**Authors:** Yao-Can Xu, Kai-Hua Chen, Zhong-Guo Liang, Xiao-Dong Zhu

**Affiliations:** ^1^ Department of Radiation Oncology, Affiliated Tumor Hospital of Guangxi Medical University, Nanning, China; ^2^ Department of Oncology, Affiliated Wu-Ming Hospital of Guangxi Medical University, Nanning, China

**Keywords:** stage II, nasopharyngeal carcinoma, chemotherapy, radiotherapy, meta-analysis

## Abstract

**Purpose:**

The role of concurrent chemoradiotherapy (CCRT) in stage II nasopharyngeal carcinoma (NPC) is still controversial. Our objective is to evaluate the value of concurrent chemotherapy in stage II NPC receiving radiotherapy (RT).

**Methods:**

We searched the PubMed, Embase, and Scopus databases for studies comparing CCRT versus RT alone in stage II NPC with survival outcomes and toxicities, including locoregional recurrence-free survival (LRFS), metastasis-free survival (DMFS), progression-free survival (PFS), overall survival (OS), and grade 3–4 acute toxicities. The hazard ratios (HRs) of survival outcomes and risk ratios (RRs) of toxicities were extracted for meta-analysis. Subgroup analysis for stage N1 patients was performed to further explore whether these populations can earn benefits from concurrent chemotherapy.

**Results:**

Nine eligible studies with a total of 4,092 patients were included. CCRT was associated with a better OS (HR = 0.61, 95% CI 0.44–0.82), LRFS (HR = 0.62, 95% CI 0.50–0.78), and PFS (HR = 0.65, 95% CI 0.54–0.79), but with similar DMFS (HR = 0.81, 95% CI = 0.46–1.45) compared with two-dimensional RT (2DRT) alone. However, CCRT showed no survival benefit in terms of OS (HR = 0.84, 95% CI 0.62–1.15), LRFS (HR = 0.85, 95% CI 0.54–1.34), DMFS (HR = 0.96, 95% CI 0.60–1.54), and PFS (HR = 0.96, 95% CI 0.66–1.37) compared with intensity-modulated RT (IMRT) alone. Subgroup analyses indicated that CCRT had similar OS (HR = 1.04, 95% CI 0.37–2.96), LRFS (HR = 0.70, 95% CI 0.34–1.45), DMFS (HR = 1.03, 95% CI 0.53–2.00), and PFS (HR = 1.04, 95% CI 0.58–1.88) in the stage N1 populations. Meanwhile, compared to RT alone, CCRT significantly increased the incidence of grade 3–4 leukopenia (RR = 4.00, 95% CI 2.29–6.97), mucositis (RR = 1.43, 95% CI 1.16–1.77), and gastrointestinal reactions (RR = 8.76, 95% CI 2.63–29.12). No significant differences of grade 3–4 toxicity in thrombocytopenia (RR = 3.45, 95% CI 0.85–13.94) was found between the two groups.

**Conclusion:**

For unselected patients with stage II NPC, CCRT was superior to 2DRT alone with better LRFS, PFS, and OS, while adding concurrent chemotherapy to IMRT did not significantly improve survival but exacerbated acute toxicities.

**Systematic Review Registration:**

https://www.crd.york.ac.uk/PROSPERO/, identifier CRD42022318253.

## Background

Nasopharyngeal carcinoma (NPC) is one of the major cancers within Southeastern Asia (1), with an annual incidence rate of 10 to 30 per 100,000 among these prevalence regions (2). Over 20% of patients present with stage II NPC at initial diagnosis (3). Radiotherapy (RT) is the main radical treatment for NPC and has brought outstanding disease control (4). Studies have shown that chemotherapy played a significant role in stage III–IVA patients (5, 6), while stage I patients cannot earn benefits from concurrent chemotherapy (7). However, the role of concurrent chemotherapy in stage II NPC remains controversial.

There are two small-sample prospective studies (8, 9) comparing concurrent chemoradiation (CCRT) with RT alone in stage II NPC patients. Among these two studies, the study (9) using two-dimensional radiotherapy (2DRT) technology reached positive results with better 10-year metastasis-free survival (DMFS), progression-free survival (PFS), overall survival (OS), and cancer-specific survival (CSS), in the CCRT group, while the other study (8) using IMRT technology obtained negative results with no survival benefit but higher hematological toxicity. However, multiple retrospective studies that compared CCRT with 2DRT alone or IMRT alone showed opposite results. Xu et al. (10) found that, compared with 2DRT, CCRT had no role in improving OS, DMFS, and PFS in stage II NPC patients, but it increased the incidence of acute adverse events. Ahmed et al. (11) reported that CCRT was superior to IMRT alone with significant benefits in OS. A systematic review (12) on treatment patterns for stage II NPC indicated that IMRT alone may be sufficient, but more aggressive treatment interventions may be needed for the T2N1M0 subgroup which has poorer survival outcomes than those in the T1N1M0 or T2N0M0 subgroup. In addition, there are three meta-analyses (13–15) evaluating the role of chemotherapy adding to RT alone for stage II NPC. Regrettably, patients with stage I/III or receiving CCRT combined with induction chemotherapy (IC) or adjuvant chemotherapy (AC) were included. The actual value of concurrent chemotherapy adding to RT is still uncertain. Therefore, we performed this meta-analysis to evaluate the benefit of concurrent chemotherapy on stage II NPC patients receiving RT.

We present the following article in accordance with the PRISMA reporting checklist (16) (**eTable 1 in the Supplement**).

## Methods

### Search Strategy

A systematic electronic search of PubMed, Embase, and Scopus databases was performed for literature published from January 1, 1990, to December 20, 2021. The detailed search strategy is presented in **eTable 2 in the Supplement**. Furthermore, we also searched relevant studies registered on ClinicalTrials.gov. A manual search of reference lists from all available reviews was conducted to identify the ultimate selection. Two investigators (Y-CX and Z-GL) independently carried out the literature retrieval.

### Selection Criteria

Studies that met the following preset specific criteria were included: (a) original English articles published in peer-reviewed journals; (b) studies that compared CCRT versus radiotherapy alone in stage II NPC patients; and (c) studies must contain time-to-event data such as locoregional recurrence-free survival (LRFS), PFS, DMFS, or OS, which could be obtained directly from the article or extracted indirectly through the method introduced by Tierney et al. (17). The LRFS was the time from the date of diagnosis to the date of first local and/or regional failure. The DMFS was considered as the interval from the date of the diagnosis to the date of distant metastasis. The PFS was defined as the interval from the date of the diagnosis to disease progression. The OS was defined as the duration from the date of diagnosis to the date of death for any reason. The exclusion criteria were as follows: (a) conference abstracts, case reports, and reviews and (b) studies involving patients who received IC and AC.

### Data Extraction and Literature Quality Assessment

Two investigators (Y-CX and Z-GL) evaluated the relevant articles according to the eligible criteria independently then extracted OS, DMFS, LRRFS, PFS, and grade 3–4 acute toxicity (leukopenia, thrombocytopenia, mucositis, gastrointestinal reactions) data from the included article, evaluated the quality of the included literature, and cross-checked the extracted data. Disagreements were resolved through discussion among the two investigators or consulting a third researcher (K-HC) to reach an agreement.

The quality of randomized controlled trial (RCT) was evaluated using the revised Cochrane risk-of-bias tool for randomized trials (RoB2) (18). The tool evaluates the risk of bias in individual RCT based on six domains: the randomization process, deviations from intended interventions, missing outcome data, measurement of the outcome, and selection of the reported result. Overall bias will be considered as low risk of bias, some concerns, or high risk of bias. Any domain-level judgement reaching a high risk of bias will result in overall high risk of bias. Some concerns for any individual domain will eventually contribute to the overall evaluation of the paper being identified as some concerns or high risk of bias. The quality of retrospective studies was assessed by the modified Newcastle–Ottawa scale assessment criteria, which comprises eight items: representativeness of the exposed cohort, selection of the non-exposed cohort, ascertainment of exposure, a demonstration that outcome of interest was not present at the start of the study, comparability of cohorts on the basis of the design or analysis, assessment of outcome, if follow-up was longer enough for outcomes to occur, and adequacy of follow-up of cohorts.

### Statistical Analysis

This meta-analysis was performed with Review Manager 5.3 software. To assess survival outcomes (OS, DMFS, LRRFS, PFS) and grade 3–4 acute toxicities (leukopenia, thrombocytopenia, mucositis, gastrointestinal reactions) between CCRT and RT alone, the HRs and relative ratios (RRs) with 95% CIs were pooled, respectively. Heterogeneity between included studies was assessed with the χ² heterogeneity test. I^2^ values of 25%, 50%, and 75% were considered as low, moderate, and high heterogeneity, respectively. The fixed-effect model was employed for meta-analysis if the heterogeneity test revealed no important heterogeneity between studies (*P* > 0.10, *I* ^2^ < 50%); otherwise, the random-effect model was applied. When the HR or RR was less than 1, it indicated a better survival outcome or safety in the CCRT group. If the 95% CI did not contain the value 1, it suggested that there was a significant difference in the statistics. Sensitivity analysis was applied to assess the stability of the survival results.

According to the Cochrane Handbook for Systematic Reviews of Interventions, we did not assess publication bias because only nine studies were included in the meta-analysis, and it was not possible to assess publication bias employing a funnel plot.

## Results

### Characteristics and Quality of Included Studies

**Figure 1:**
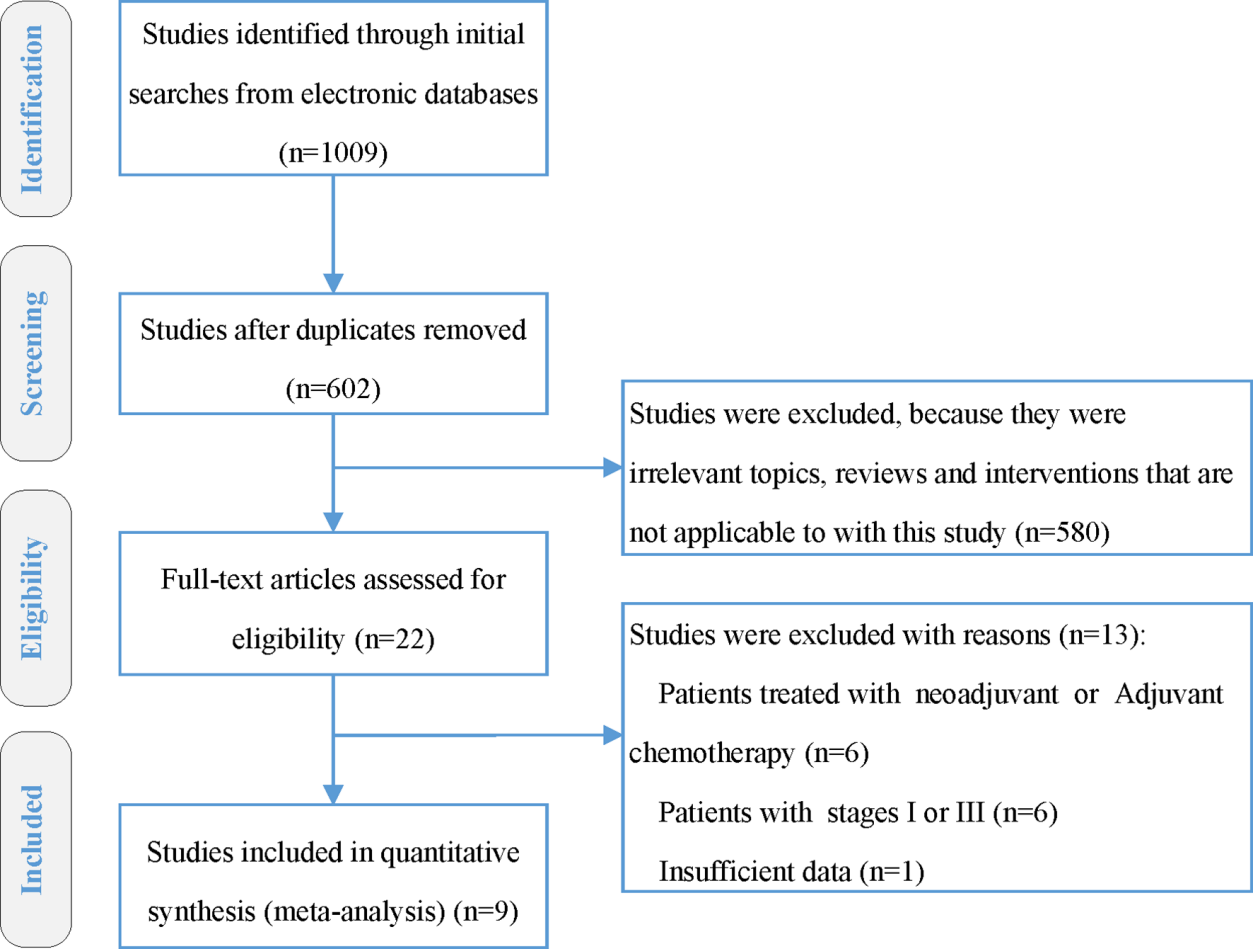
PRISMA flow diagram.

**Figure 2:**
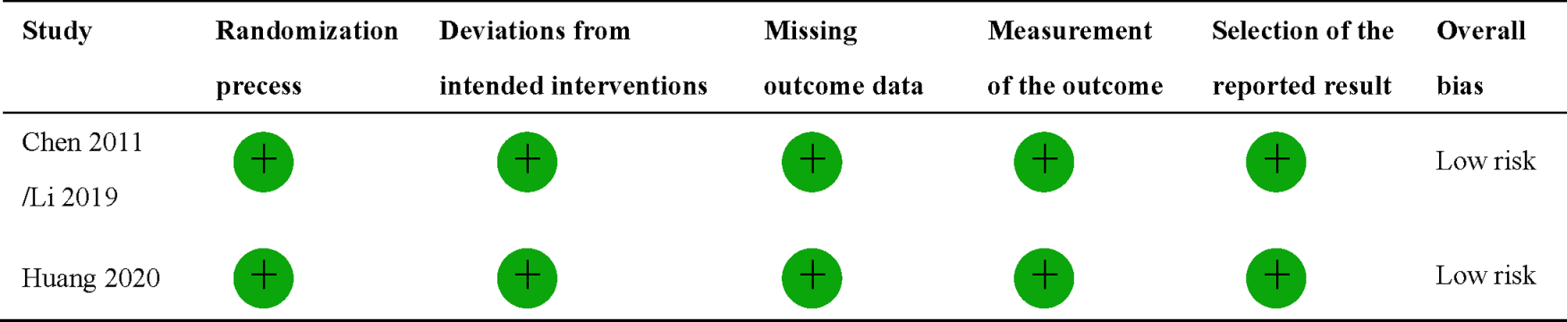
Assessment of quality of randomized controlled trials.

Totally 1,009 items, including 435 from PubMed, 287 from Embase, and 287 from Scopus, were obtained after the initial search. After duplication removal, 602 studies were retrieved. Only 22 studies remained after the titles and abstracts were assessed. Among the remaining 22 studies, six studies (19–24) involving patients with adjuvant or neoadjuvant chemotherapy were excluded, another six studies (25–30) involving patients with stage I or III were eliminated, and one study (31) with insufficient data was also eliminated **(** **Figure 1** **)**. Nine studies were finally included, two of which were RCTs (8, 9), and the rest were retrospective studies (10, 11, 32–36). A total of 4,092 patients were enrolled, 2,462 received CCRT, and 1,632 received RT alone. There are four studies (9, 10, 32, 34) with a total of 2,490 patients that investigated 2DRT combined with concurrent chemotherapy, and 7 studies (8, 11, 32–36) with 1,602 patients that explored IMRT plus concurrent chemotherapy (**Table 1**). According to RoB2 assessment criteria, the overall risk of bias was low for the two included RCTs (**Figure 2**). According to the Newcastle–Ottawa Scale assessment criteria, four retrospective studies received eight stars, and the other three got nine stars (**Table 2**).

**Table 1:** Eligible study characteristics.

Study	Study design	No. of patients (CCRT/RT)	Inclusion period	Stage	Median follow-up (months)	Radiotherapy	Concurrent chemotherapy
Li 2021 (34)	R	2DRT: 348 (159/189) IMRT: 253 (96/157)	2003–2016	AJCC-2010 II	2DRT:103.0 IMRT: 99.0	2DRT: T66–70 Gy, N+60–62 Gy, N-50 Gy IMRT: T66–70 Gy, N+66–70 Gy	Cisplatin or nedaplatin 35 mg/m^2^, qw or 80–100 mg/m2, q3w
Chen 2011 (37)/Li 2019 (9)	RCT	230 (116/114)	2003–2007	Chinese-1992 II	125.0	2DRT: T68–70 Gy, N+60–62 Gy, N-50 Gy	Cisplatin 30 mg/m^2^, qw
Xu 2015 (35)	R	86 (43/43)	2009–2011	AJCC-2002 II	37.4	IMRT: T66 Gy, N+60 Gy, N-54 Gy	Cisplatin 40 mg/m^2^, qw
Jin 2021 (36)	R	354 (177/177)	2008–2016	AJCC-2017 II	69.9	IMRT: T66–72 Gy, N+64–70Gy, N-54–56 Gy	Cisplatin 40 mg/m^2^, qw, or 80 mg/m^2^, q3w
Ahmed 2019 (11)	R	172 (116/56)	2004–2013	AJCC-2010 II	50.4	IMRT: T66–70 Gy	NR
Liu 2020 (32)	R	2DRT: 1520 (304/1216) IMRT: 404 (202/202)	1990–2012	AJCC-2010 II	2DRT: 93 IMRT: 44.0	2DRT: T66–72 Gy, IMRT: T66–72 Gy	Cisplatin 30–40 mg/m^2^ qw, or 80–100 mg/m^2^ q3w
Xu 2011 (10)	R	392 (181/211)	2000–2003	AJCC-2002 II	66.0	2DRT: T70 Gy, N+66–70 Gy	Cisplatin 100 mg/m^2^, q3w
Su 2016 (33)	R	24 9(143/106)	2005–2010	AJCC-2010 II	59.4	IMRT: T66–70 Gy, N+60–64 Gy, N-42–62 Gy	platinum single-agent (qw or q3w), paclitaxel, TP or PF
Huang 2020 (8)	RCT	84 (41/43)	2010–2012	AJCC-2010 II	75.0	IMRT: T69.96 Gy, N+60.06 Gy, N-50.96 Gy	Cisplatin 40 mg/m^2^, qw

CCRT, concurrent chemoradiation; 2D-RT, two-dimensional radiotherapy; IMRT, intensity-modulated radiotherapy; AJCC, American Joint Committee on Cancer; R, retrospective; RCT, randomized controlled trial; NR, not reported.

**Table 2:** Assessment of quality of non-randomized studies.

Study	Selection				Comparability	Outcome			Score
	Representativeness of the exposed cohort	Selection of the non-exposed cohort	Ascertainment of exposure	Demonstration that outcome of interest was not present at start of study	Comparability of cohorts on the basis of the design or analysis	Assessment of outcome	Was follow-up longer enough for outcomes to occur	Adequacy of follow-up of cohorts	
Li 2021 (34)	*	*	*	*	*	*	*	*	8
Xu 2015 (35)	*	*	*	*	*	*	*	*	8
Jin 2021 (36)	*	*	*	*	**	*	*	*	9
Ahmed 2019 (11)	*	*	*	*	**	*	*	*	9
Liu 2020 (32)	*	*	*	*	**	*	*	*	9
Xu 2011 (10)	*	*	*	*	*	*	*	*	8
Su 2016 (33)	*	*	*	*	*	*	*	*	8

“*” represents the score of each item in the evaluation criteria (full score of “Selection” is 4 points, full score of "Comparability" is 2 points, full score of "Outcome" is 3 points. The higher the score is, the higher the quality of the paper is), "*" represents 1 point, "**" represents 2 points.

### Survival Outcomes

Six studies directly provided HR values and 95%CI of time-to-event data, and the other three studies (8, 10, 33) did not provide HR values but provided survival curves. The method recommended by Tierney was used to extract HR and 95% CI from survival curves. OS data were available in all included studies.

**Figure 3:**
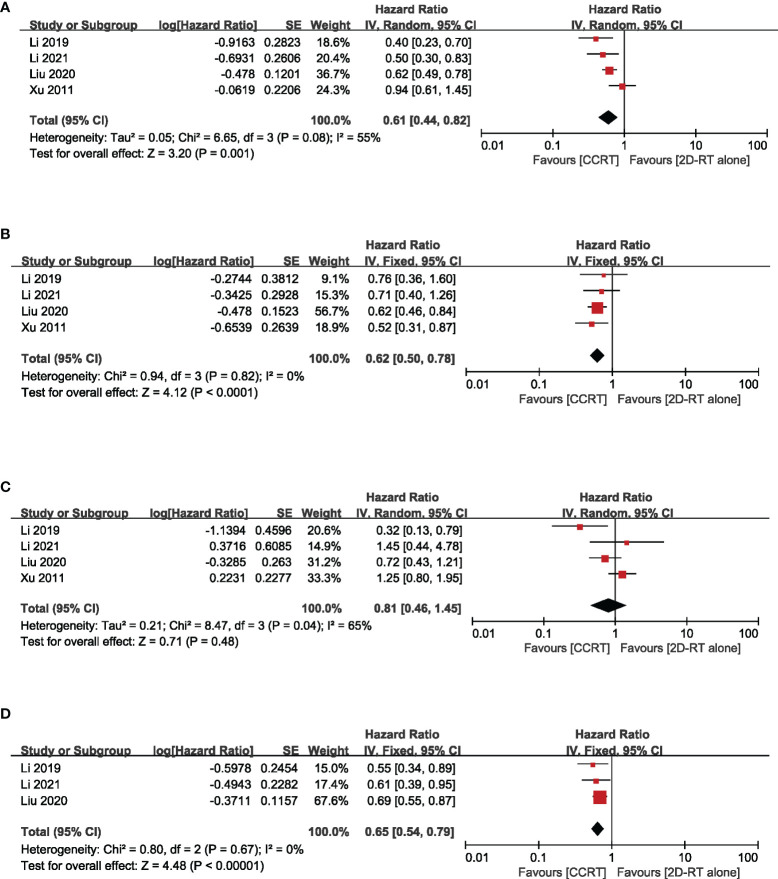
Forest plot of the meta-analysis regarding OS **(A)**, LRFS **(B)**, DMFS **(C)**, and PFS **(D)** with CCRT vs. 2DRT alone. OS, overall survival; LRFS, locoregional recurrence-free survival; DMFS, distant metastasis-free survival; PFS, progression-free survival; CCRT, concurrent chemoradiation; 2D-RT, two-dimensional radiotherapy.

**Figure 4:**
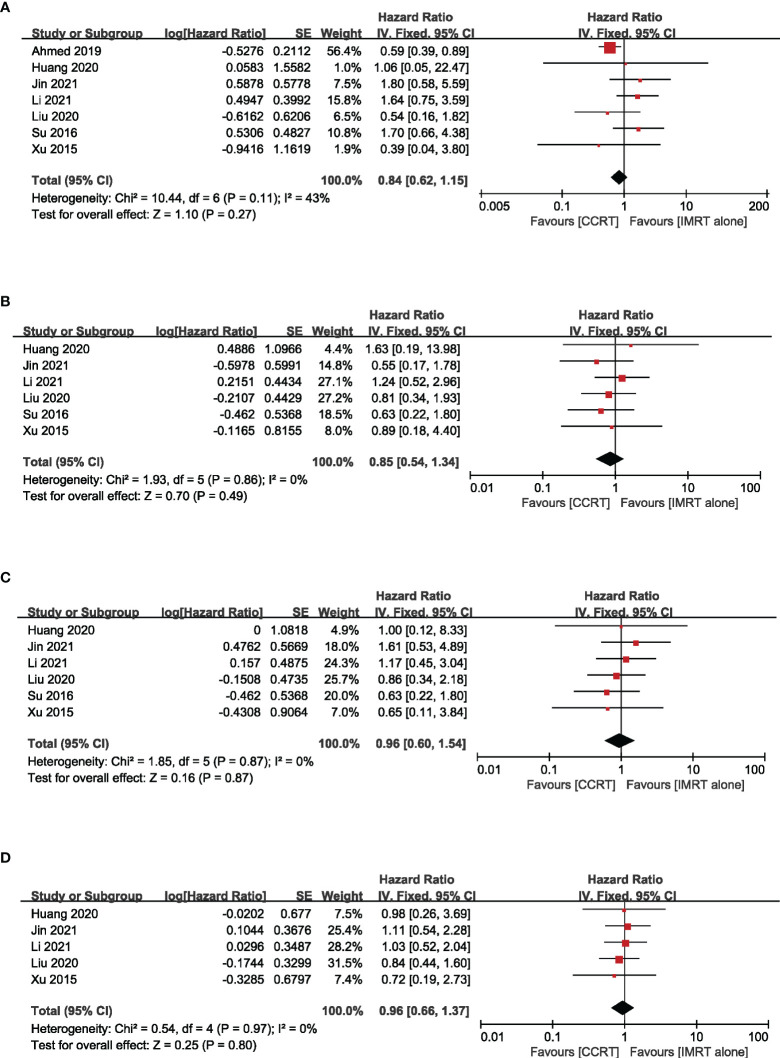
Forest plot of the meta-analysis regarding OS **(A)**, LRFS **(B)**, DMFS **(C)**, and PFS **(D)** with CCRT vs. IMRT alone. IMRT, intensity-modulated radiotherapy.

**Figure 5:**
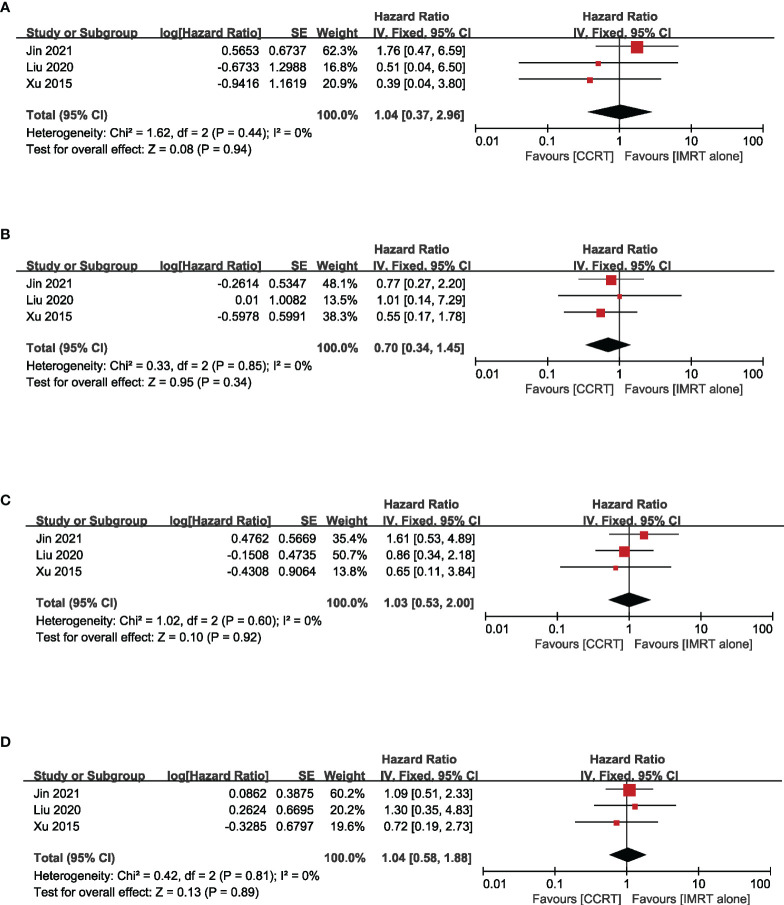
Forest plot of the meta-analysis regarding OS **(A)**, LRFS **(B)**, DMFS **(C)**, and PFS **(D)** with CCRT vs. IMRT alone in the N1 subgroup.

Based on different radiotherapy techniques, the included studies were separated into two categories and meta-analyses were performed respectively. It revealed that, for stage II NPC patients undergoing 2DRT, concurrent chemotherapy could significantly prolong OS (HR = 0.61, 95% CI 0.44–0.82) (heterogeneity *P* = 0.08, *I* ^2^ = 55%), LRFS (HR = 0.62, 95% CI 0.50–0.78) (heterogeneity *P* = 0.82, *I* ^2^ = 0.00%), and PFS (HR = 0.65, 95% CI 0.54–0.79) (heterogeneity *P* = 0.67, *I* ^2^ = 0.00%), except DMFS (HR = 0.81, 95% CI = 0.46–1.45) (heterogeneity *P* = 0.04, *I* ^2^ = 65%) (**Figure 3**). Nevertheless, with IMRT, no remarkable difference between the CCRT group and the IMRT-alone group was observed in terms of OS (HR = 0.84, 95% CI 0.62–1.15) (heterogeneity *P* = 0.11, *I* ^2^ = 43%), LRFS (HR=0.85, 95% CI 0.54–1.34) (heterogeneity *P* = 0.86, *I* ^2^ = 0.00%), DMFS (HR = 0.96, 95% CI 0.60–1.54) (heterogeneity *P* = 0.87, *I* ^2^ = 0.00%), and PFS (HR = 0.96, 95% CI 0.66–1.37) (heterogeneity *P* = 0.97, *I* ^2^ = 0.00%) (**Figure 4**). Moreover, to explore the potential beneficiaries of concurrent chemotherapy for stage II NPC in the IMRT era, we conducted a subgroup analysis of stage T1-2N1 patients treated with IMRT. Unfortunately, it was found that additional concurrent chemotherapy did not improve OS (HR = 1.04, 95% CI 0.37–2.96) (heterogeneity *P* = 0.44, *I* ^2^ = 0.00%), LRFS (HR = 0.70, 95% CI 0.34–1.45) (heterogeneity *P* = 0.85, *I* ^2^ = 0.00%), DMFS (HR = 1.03, 95% CI 0.53–2.00) (heterogeneity *P* = 0.60, *I* ^2^ = 0.00%), and PFS (HR = 1.04, 95% CI 0.58–1.88) (heterogeneity *P* = 0.81, *I* ^2^ = 0.00%) in this population (**Figure 5**).

### A Sensitivity Analysis

The stability of the results was evaluated by removing some studies according to different standards (**Table 3**). First of all, the sensitivity analysis was conducted in IMRT studies by separately eliminating two studies (8, 35) with a sample size of less than 100 patients, four studies (11, 32, 33, 35) with a median follow-up time of fewer than 60 months, and three studies (11, 33, 34) that included concurrent chemotherapy regimens other than cisplatin, respectively. It suggested that OS, LRFS, DMFS, and PFS were similar between the CCRT group and IMRT alone group, which was consistent with that before sensitivity analysis. Then, the sensitivity analysis was carried out in 2DRT studies by excluding one study that included concurrent chemotherapy with nedaplatin. There was no statistically significant change in survival outcomes. We did not perform sensitivity analyses for sample size and follow-up time because all 2DRT studies had a sample size of more than 100 and were followed up for more than 60 months. In summary, the survival results of the meta-analysis were robust and reliable.

**Table 3:** Sensitivity analysis for the comparison of CCRT and RT alone.

Outcome	Patients		Effect	*P*-value	Heterogeneity			*P*-value
	CCRT	RT alone	HR (95% CI)		χ^2^	*df*	*I* ^2^ (%)	
**IMRT**								
Sample size >100 patients								
OS	734	698	1.05 (0.58–19.98)	0.87	9.98	4	60%	**0.04**
LRFS	618	642	0.82 (0.51–1.33)	0.42	1.56	3	0%	0.67
DMFS	618	642	0.99 (0.60–1.64)	0.97	1.65	3	0%	0.65
PFS	475	536	0.98 (0.66–1.45)	0.91	0.35	2	0%	0.84
Median follow-up time > 60 months								
OS	314	377	1.66 (0.88–3.11)	0.12	0.10	2	0%	0.95
LRFS	314	377	0.98 (0.50–1.91)	0.96	1.43	2	0%	0.49
DMFS	314	377	1.30 (0.65–2.58)	0.45	0.25	2	0%	0.88
PFS	314	377	1.06 (0.66–1.68)	0.82	0.25	2	0%	0.98
Concurrent chemotherapy with cisplatin								
OS	463	465	0.93 (0.44–1.97)	0.84	2.64	3	0%	0.45
LRFS	463	465	0.78 (0.42–1.44)	0.43	0.83	3	0%	0.84
DMFS	463	465	1.03 (0.55–1.91)	0.93	1.02	3	0%	0.80
PFS	463	465	0.93 (0.60–1.42)	0.73	0.47	3	0%	0.92
**2DRT**								
Concurrent chemotherapy with cisplatin								
OS	601	1743	0.63 (0.43–0.94)	**0.02**	5.88	2	66%	**0.05**
LRFS	601	1743	0.61 (0.48–0.78)	**<0.0001**	0.71	2	0%	0.70
DMFS	601	1743	0.73 (0.37–1.42)	0.35	7.78	2	74%	**0.02**
PFS	601	1736	0.66 (0.54–0.81)	**<0.0001**	0.70	1	0%	0.40

CCRT, concurrent chemoradiation; 2D-RT, two-dimensional radiotherapy; IMRT, intensity-modulated radiotherapy; HR, hazard ratio; CI, confidence interval; df, degrees of freedom; OS, overall survival; LRFS, locoregional recurrence-free survival; DMFS, distant metastasis-free survival; PFS, progression-free survival.P values less than 0.05 are shown in bold (bold numbers), indicating that the therapeutic effect or heterogeneity of the included literature is statistically significant.

### Acute Toxicity

**Figure 6:**
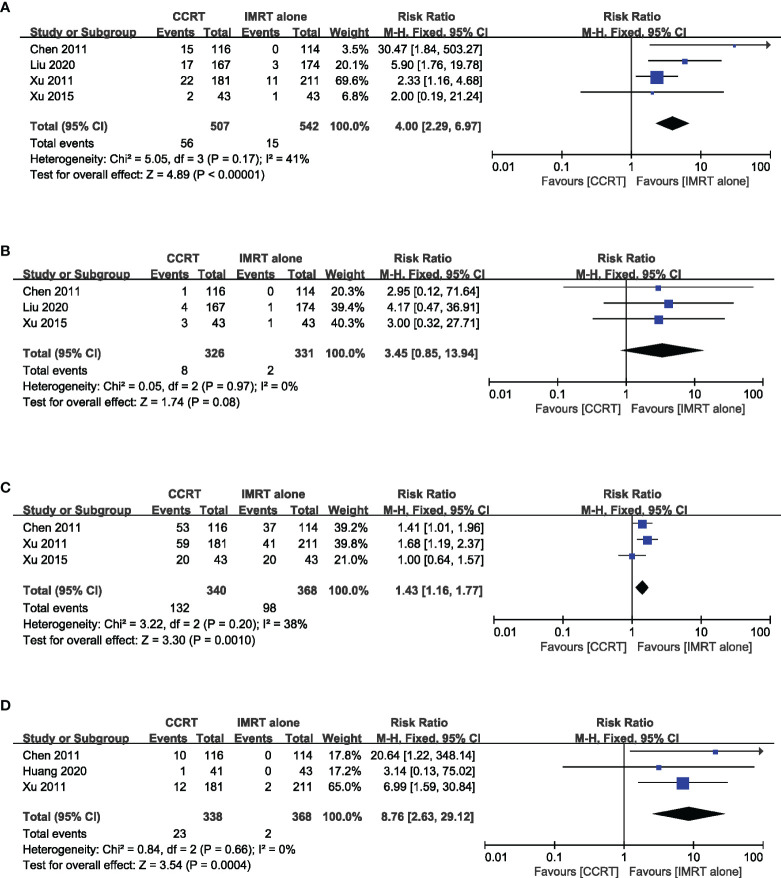
Forest plot of the meta-analysis regarding grade 3–4 leukopenia **(A)**, thrombocytopenia **(B)**, mucositis **(C)**, and gastrointestinal reactions **(D)** with CCRT vs. IMRT alone.

The incidence of grade 3–4 acute toxicity was reported in five studies with a total of 741 patients. The results of the meta-analysis suggested that the incidence of grade 3–4 leukopenia (RR = 4.00, 95% CI 2.29~6.97) (heterogeneity *P* = 0.14, *I* ^2^ = 41%), mucositis (RR = 1.43, 95% CI 1.16–1.77) (heterogeneity *P* = 0.20, *I* ^2^ = 38%), and gastrointestinal reaction (RR = 8.76, 95% CI 2.63~29.12) (heterogeneity *P* = 0.66, *I* ^2^ = 0%) in the CCRT group were significantly higher than those in the IMRT-alone group. The incidence of grade 3–4 thrombocytopenia (RR = 3.45, 95% CI 0.85–13.94) (heterogeneity *P* = 0.97, *I* ^2^ = 0%) was similar in the two groups (**Figure 6**).

## Discussion

Radiotherapy is the main treatment for NPC. Stage III–IVA NPC patients receiving CCRT can further earn survival benefits from induction chemotherapy (5), but so far, whether chemotherapy can bring survival benefits to stage II patients is still controversial. The ASCO and CSCO Guideline recommends that it is not necessary for stage II NPC to routinely receive chemotherapy unless there are high-risk factors, such as high pretreatment EBV-DNA level, bulky tumor volumes, or extranodal extension (38). We assessed the therapeutic effect and toxicity of CCRT compared with 2DRT alone or IMRT alone for stage II NPC patients by conducting a meta-analysis.

Our study suggested that, compared with 2DRT alone, CCRT was associated with improved OS, LRFS, and PFS in stage II NPC patients. In the 2DRT era, a retrospective study conducted by Cheng and colleagues (23) revealed that stage II NPC patients receiving CCRT had similar PFS and LRFS compared with stage I patients receiving 2DRT alone. Another large retrospective study (39) included 1,790 patients and exhibited that the N1 subgroup of stage II NPC patients is more prone to distant metastasis, leading to a poorer prognosis. Furthermore, a combined subgroup analysis from two RCTs showed the survival benefit obtained from two or three cycles of cisplatin-based induction chemotherapy in stage II NPC (40). Hence, for stage II IPC patients treated with 2DRT, concurrent chemotherapy is highly crucial, particularly for the T1-2N1 population.

IMRT has become a daily choice for NPC. Several studies (8, 11, 32–36) have investigated whether concurrent chemotherapy can further improve the efficacy of stage II NPC patients receiving IMRT. However, the results of these studies are not completely consistent. Our meta-analysis revealed that concurrent chemotherapy had no therapeutic effect but increased toxicity in patients with stage II NPC receiving IMRT. Multiple possible explanations could account for the negative result in survival outcomes. First of all, as a high-precision radiotherapy therapy, IMRT can not only accurately irradiate the irregular tumor target with a higher dose but also protect the adjacent critical structures to the greatest extent. Several studies (41, 42) have consistently found that IMRT can significantly reduce radiation-induced toxicity and improve local control and long-term survival outcomes versus 2DRT, particularly for T1-2 patients (26, 41, 43). A prospective randomized study (41) comparing 2DRT with IMRT suggested that, with 2DRT, the 5-year OS and local control rates of stage II NPC were 67.1% and 84.7%, respectively, while with IMRT, they can be increased to 79.6% and 90.5%, respectively. Lai and colleagues (44) performed a retrospective study and found that IMRT significantly prolonged 5-year LRFS for patients with stage II NPC (92.7% vs. 86.8%) compared with 2DRT. The 5-year LRFS of stage T1 patients even reached 100% in the IMRT group versus 94.4% in the 2DRT group. Interestingly, a study (26) directly comparing IMRT alone with 2DRT plus concurrent chemotherapy indicated that the two groups were similar in terms of 4-year OS, LRFS, and DMFS (97.4% vs. 97.4%; 93.8% vs. 95.7%; 96.5% vs. 97.3%, respectively). Thanks to the progress of radiation therapy technology, the 5-year OS and local control rates of stage II NPC have improved substantially in the IMRT era. Concurrent chemotherapy might not bring survival benefits to this population. Secondly, an update result from the only phase 3 RCT (9) for stage II NPC revealed that CCRT significantly improved the 10-year OS (83.6% vs. 65.8%) and PFS (76.7% vs. 64.0%) compared to RT alone. However, the enrolled patients were evaluated by the Chinese 1992 staging system, and 31 (13%) of them were reclassified as stage III/N2 based on the AJCC TNM Staging System (7th ed., 2017). The survival benefit from stage N2 patients may lead to an overestimation of the role of concurrent chemotherapy in this study. Thirdly, stage II NPC is composed of three subsets (T2N0, T1N1, and T2N1), with obvious heterogeneity. Each subgroup has a different prognosis, and N1 patients are more likely to develop distant metastases (39). Hence, we conducted an N1 subgroup analysis for stage II patients. Unfortunately, it was found that additional concurrent chemotherapy did not improve survival outcomes.

Studies have found that baseline characteristics, such as plasma EBV-DNA level (45), lymph node size (46), and extranodal extension (47, 48), were independent unfavorable factors of NPC. Growing evidence indicated that the plasma EBV-DNA level was highly associated with tumor burden and elevated pretreatment plasma EBV-DNA was related to worse clinical outcomes (49, 50). EBV DNA-positive stage II patients had similar overall survival to stage III patients (51). This is the first study to demonstrate that pretreatment plasma EBV-DNA can be used to distinguish high-risk subgroups in early-stage patients. Results from real-world research (52) indicated that high pretreatment plasma EBV-DNA levels (≥4,000 copies/ml) was an adverse independent factor in LA-NPC. Patients with high EBV-DNA levels had a comparable survival outcome to T4 or N2–3 patients, with a 5-year PFS of 69%. Another large cohort study (53) of 1,357 patients with LA-NPC revealed that, for patients with high EBV-DNA levels (>4,000 copies/ml), IMRT with concurrent chemotherapy improved OS, DFS, and DMFS compared with IMRT alone. However, there was no observed benefit with the addition of concurrent chemotherapy in patients with low EBV-DNA levels. Pretreatment EBV-DNA has been widely accepted as a useful prognostic biomarker and plays an important role in tailoring treatment strategies in the clinic (54). Therefore, stage II NPC patients with high pretreatment EBV-DNA levels might be ideal candidates for concurrent chemotherapy. However, two issues need to be addressed before EBV-DNA was widely used in clinical practice for risk stratification. Firstly, the harmonization and standardization of the quantitative plasma EBV-DNA measurement between laboratories have not been established, resulting in poor inter-laboratory concordance. Secondly, although the EBV-DNA cutoff values have been set at 2,000 or 4,000 copies/ml in most studies, there is still no consensus on the optimal thresholds for risk discretization. A retrospective study showed that the tumor volume was a significant independent predictor of increasing risk of recurrence (33). Another study (55) from Hong Kong reported no role of using concurrent chemotherapy in stage II NPC, except for lymph nodes >2 cm. However, these two studies are small-sample retrospective studies, and the value of tumor volume and lymph node size needs to be further studied. Studies demonstrated that extranodal extension played an important role in predicting distant metastasis in stage II NPC patients with N1 category (56–58). Patients with high-grade extranodal extension (including coalescent nodes and metastatic node infiltrating into adjacent structures) had a significantly higher risk of distant metastasis and death than those without (including metastatic nodes infiltrating into surrounding fat and without extranodal extension) and were suggested to be classified as cN3. However, patients with metastatic nodes infiltrating into surrounding fat (low-grade extranodal extension) had a similar outcome to those without extranodal extension. Hence, stage II nasopharyngeal carcinoma patients with high-grade extranodal invasion are likely candidates for concurrent chemotherapy. Although the risk stratification factors mentioned above might have the potential to identify candidates for concurrent chemotherapy, we were unable to conduct further subgroup analyses for these factors because they were not reported in the included literature. The tumor volume, size of metastatic lymph nodes, extranodal extension, and EBV-DNA levels were not disaggregated in the N1 subgroup analysis, which may have a significant impact on the results. Therefore, the negative results of the N1 subgroup analysis in this study should be interpreted with caution. Future studies should focus on these high-risk groups who are most likely to benefit from chemotherapy.

Stage II NPC has a good prognosis, with 5-year OS 97.8%, so it is particularly significant to relieve toxicity and improve quality of life (59). Studies in terms of anti-EGFR antibodies, such as cetuximab, nimotuzumab, and Endostar, combined with RT in patients with LA-NPC have been launched. Xu and colleagues carried out a comparative study between concurrent cisplatin-chemoradiotherapy (CRT) and cetuximab-radiotherapy (ERT) (60). ERT was not superior to CRT, while it was more prone to result in acute adverse events. Similar results were obtained in another retrospective study (61); cetuximab/nimotuzumab combined concurrently with IMRT suggested equivalence to the standard CCRT in terms of DFS, LRRFS, DMFS, and OS. Skin reaction and mucositis are more common in the cetuximab/nimotuzumab group. A phase II study enrolling 23 stage III–IV NPC patients found that, compared to CCRT, radiotherapy combined with Endostar had similar efficacy, but lighter acute adverse reactions, which improved quality of life (62). In conclusion, Endostar has the potential to serve as a concurrent treatment option for the high-risk subgroup of stage II patients and deserves further study. Anti-PD1 checkpoint inhibitors, such as nivolumab (63), pembrolizumab (64), camrelizumab (65, 66), toripalimab (67), and tislelizumab (68), had a clinically meaningful antitumor activity with a manageable safety profile. Two phase 3 trials demonstrated that, as first-line treatment for recurrent/metastatic NPC, camrelizumab or toripalimab in combination with gemcitabine and cisplatin prolonged PFS as compared to gemcitabine and cisplatin (median PFS 9.7 vs. 6.9 months, 11.7 vs. 8.0 months, respectively) (65, 67). Several phase II–III trials (NCT05305131, NCT03700476, NCT03267498, NCT04782765, NCT04227509, NCT03427827, NCT04557020, NCT04453826, NCT05211232) are in progress to clarify the efficacy and safety of PD-1 in combination with CCRT for high-risk LA-NPC (except for T3N0–1 and T4N0). Of particular concern is a phase II trial (NCT05229315) evaluating the safety and efficacy of toripalimab combined with IMRT in the treatment of stage II NPC. Nevertheless, risk stratification factors, such as EBV-DNA, lymph node size, and extranodal extension, were not evaluated as a part of eligibility criteria in most studies, with the exceptions of NCT04453826 (enrolled patients were required to have EBV-DNA >0 copies/ml after 3 cycles of induction chemotherapy) and NCT05229315 (enrolled patients were required to have EBV-DNA <4,000 copies/ml).

In terms of acute toxicities, this meta-analysis found that grade 3–4 leukopenia, mucositis, and gastrointestinal reactions were more frequent in patients receiving CCRT versus IMRT alone. A previous study (69) suggested that CCRT is related to higher incidences of treatment-related mortality (1.7% vs. 0.8%) as compared with radiotherapy alone. Leukopenia is the most common cause of death. Because of higher acute toxicity and treatment-related death, the application of concurrent chemotherapy in stage II NPC should be considered prudently. Currently, four RCTs (NCT02116231, NCT02633202, NCT02610010, NCT03068936) that evaluate the role of concurrent chemotherapy for stage II patients are ongoing in China, and the eventual results are expected to be released in the near future.

The present meta-analysis has multiple limitations. First of all, both RCTs and retrospective studies were enrolled, which may influence the level of evidence to some extent. Second, because staging systems vary in some included studies, it may contribute to heterogeneity in this meta-analysis. Third, several studies with relatively small sample sizes or median follow-up of less than 5 years were included. Finally, survival data of three studies (8, 10, 33) were obtained from survival curves by Tierney’s methods, which may lead to potential bias.

## Conclusion

In summary, for patients with stage II NPC, current evidence suggested that CCRT was superior to 2DRT alone with significantly better LRFS, PFS, and OS. However, IMRT alone was comparable to CCRT with similar efficacy but lower acute toxicities. Consequently, routine use of concurrent chemotherapy in unselected patients should not be encouraged in the IMRT era. There is an urgent need to identify subgroups of stage II patients who might derive clinical benefits from concurrent chemotherapy.

## Data Availability Statement

The original contributions presented in the study are included in the article/**Supplementary Material**. Further inquiries can be directed to the corresponding author.

## Author Contributions

Study design: X-DZ, K-HC; data acquisition: Y-CX, Z-GL; quality control of data: Y-CX, Z-GL, and K-HC; data analysis and interpretation: Y-CX, Z-GL; statistical analysis: Y-CX, Z-GL; manuscript preparation: Y-CX, Z-GL, and K-HC; manuscript review: X-DZ. All authors read and X-DZ approved the final manuscript. All authors contributed to the article and approved the submitted version.

## Conflict of Interest

The authors declare that the research was conducted in the absence of any commercial or financial relationships that could be construed as a potential conflict of interest.

## Publisher’s Note

All claims expressed in this article are solely those of the authors and do not necessarily represent those of their affiliated organizations, or those of the publisher, the editors and the reviewers. Any product that may be evaluated in this article, or claim that may be made by its manufacturer, is not guaranteed or endorsed by the publisher.
