# Supplementary material for: A Systematic Review and Meta-Analysis of Studies Comparing Concurrent Chemoradiotherapy With Radiotherapy Alone in the Treatment of Stage II Nasopharyngeal Carcinoma
Source: Front Oncol. 2022 Jul 12;12:843675. doi: 10.3389/fonc.2022.843675 (PMC9317745; doi:10.3389/fonc.2022.843675)
Supplement: Supplementary file 2 [file Table_2.docx]

| Nasopharyngeal Carcinoma | #1 | ("Nasopharyngeal Carcinoma"[Mesh]) OR (((Carcinoma, Nasopharyngeal) OR (Carcinomas, Nasopharyngeal)) OR (Nasopharyngeal Carcinomas)) |
| --- | --- | --- |
| Stage II | #2 | (Stage II[Title/Abstract]) OR (early stage [Title/Abstract]) OR (T0N1M0 [Title/Abstract]) OR (T1N1M0 [Title/Abstract]) OR (T2N0M0 [Title/Abstract]) OR (T2N1M0 [Title/Abstract]) |
| Radiotherapy | #3 | "Radiotherapy"[Mesh] OR Radiotherapies OR Radiation Therapy OR Radiation Therapies OR Therapies, Radiation OR Therapy, Radiation OR Radiation Treatment OR Radiation Treatments OR Treatment, Radiation OR Radiotherapy, Targeted OR Radiotherapies, Targeted OR Targeted Radiotherapies OR Targeted Radiotherapy OR Targeted Radiation Therapy OR Radiation Therapies, Targeted OR Targeted Radiation Therapies OR Therapies, Targeted Radiation OR Therapy, Targeted Radiation OR Radiation Therapy, Targeted |
| Chemoradiotherapy | #4 | "Chemoradiotherapy"[Mesh] OR Chemoradiotherapies OR Radiochemotherapy OR Radiochemotherapies OR Concurrent Chemoradiotherapy OR Chemoradiotherapies, Concurrent OR Chemoradiotherapy, Concurrent OR Concurrent Chemoradiotherapies OR Synchronous Chemoradiotherapy OR Chemoradiotherapies, Synchronous OR Chemoradiotherapy, Synchronous OR Synchronous Chemoradiotherapies OR Concurrent Radiochemotherapy OR Concurrent Radiochemotherapies OR Radiochemotherapies, Concurrent OR Radiochemotherapy, Concurrent OR Concomitant Chemoradiotherapy OR Chemoradiotherapies, Concomitant OR Chemoradiotherapy, Concomitant OR Concomitant Chemoradiotherapies OR Concomitant Radiochemotherapy OR Concomitant Radiochemotherapies OR Radiochemotherapies, Concomitant OR Radiochemotherapy, Concomitant |
| Combination Chemotherapy | #5 | "Drug Therapy, Combination"[Mesh] OR Combination Chemotherapy OR Drug Polytherapy OR Drug Polytherapies OR Polytherapies, Drug OR Polytherapy, Drug OR Therapy, Combination Drug OR Chemotherapy, Combination OR Chemotherapies, Combination OR Combination Chemotherapies OR Combination Drug Therapy OR Combination Drug Therapies OR Drug Therapies, Combination OR Therapies, Combination Drug OR Polychemotherapy OR Polychemotherapies |
| Language | #6 | English [Language] |
| Date publication | #7 | "1990/01/01"[Date - Publication]: "2021/12/20"[Date - Publication] |
| **Search strategy** | #8 | #3 OR #4 OR #5 |
|  | #9 | #1 AND #2 AND #6 AND #7 AND #8 |

**eTable 2. Search Strategy**
